# The Malnutrition-Related Increase in Early Visceralization of *Leishmania donovani* Is Associated with a Reduced Number of Lymph Node Phagocytes and Altered Conduit System Flow

**DOI:** 10.1371/journal.pntd.0002329

**Published:** 2013-08-15

**Authors:** Marwa K. Ibrahim, Jeffrey L. Barnes, Gregory M. Anstead, Fabio Jimenez, Bruno L. Travi, Alex G. Peniche, E. Yaneth Osorio, Seema S. Ahuja, Peter C. Melby

**Affiliations:** 1 Department of Microbiology and Immunology, University of Texas Health Science Center at San Antonio, San Antonio, Texas, United States of America; 2 South Texas Veterans Health Care System, San Antonio, Texas, United States of America; 3 Department of Medicine, University of Texas Health Science Center at San Antonio, San Antonio, Texas, United States of America; 4 Department of Internal Medicine, University of Texas Medical Branch, Galveston, Texas, United States of America; 5 Center for Tropical Diseases, University of Texas Medical Branch, Galveston, Texas, United States of America; 6 Department of Microbiology and Immunology, University of Texas Medical Branch, Galveston, Texas, United States of America; 7 Department of Pathology, University of Texas Medical Branch, Galveston, Texas, United States of America; Queensland Institute of Medical Research, Australia

## Abstract

In a murine model of moderate childhood malnutrition we found that polynutrient deficiency led to a 4–5-fold increase in early visceralization of *L. donovani* (3 days post-infection) following cutaneous infection and a 16-fold decrease in lymph node barrier function (p<0.04 for all). To begin to understand the mechanistic basis for this malnutrition-related parasite dissemination we analyzed the cellularity, architecture, and function of the skin-draining lymph node. There was no difference in the localization of multiple cell populations in the lymph node of polynutrient deficient (PND) mice, but there was reduced cellularity with fewer CD11c^+^dendritic cells (DCs), fibroblastic reticular cells (FRCs), MOMA-2^+^ macrophages, and CD169^+^ subcapsular sinus macrophage (p<0.05 for all) compared to the well-nourished (WN) mice. The parasites were equally co-localized with DCs associated with the lymph node conduit network in the WN and PND mice, and were found in the high endothelial venule into which the conduits drain. When a fluorescent low molecular weight (10 kD) dextran was delivered in the skin, there was greater efflux of the marker from the lymph node conduit system to the spleens of PND mice (p<0.04), indicating that flow through the conduit system was altered. There was no evidence of disruption of the conduit or subcapsular sinus architecture, indicating that the movement of parasites into the subcortical conduit region was due to an active process and not from passive movement through a leaking barrier. These results indicate that the impaired capacity of the lymph node to act as a barrier to dissemination of *L. donovani* infection is associated with a reduced number of lymph node phagocytes, which most likely leads to reduced capture of parasites as they transit through the sinuses and conduit system.

## Author Summary

The impact of malnutrition in the world is staggering. Malnutrition is thought to directly or indirectly contribute to more than half of all childhood deaths, most of them related to heightened susceptibility to infection. Visceral leishmaniasis (VL), caused by the intracellular protozoan *Leishmania donovani*, is a progressive, potentially fatal infection found in many resource-poor regions of the world. Most people who get infected with this parasite have only an asymptomatic latent infection, however, people who are malnourished have a greatly increased risk of developing severe VL. We initiated these studies of an experimental model that mimics human childhood malnutrition to better understand how malnutrition increases the susceptibility to VL at the molecular and cellular level. In this model we found that malnutrition led to failure of the skin-draining lymph node to act as a barrier to dissemination. This loss of lymph node barrier function was associated with a significant reduction in the numbers of dendritic cells and macrophages, phagocytic cells that capture and kill invading pathogens, and alteration of the flow of lymph through the lymph node.

## Introduction

Protein-energy malnutrition (PEM) is thought to be the most frequent cause of human immunodeficiency [1], and greatly predisposes individuals to infectious diseases in resource-poor regions of the world [2]. In its synergy with infection, under-nutrition contributes to approximately 50% of childhood deaths worldwide [3]. Apart from PEM, deficiencies in single nutrients, such as vitamins, fatty acids, amino acids and trace elements also alter immune function and increase the risk of infection [2].

Both innate and adaptive immunity may be impaired in the malnourished host [4], leading to increased susceptibility to infectious diseases [5]. Malnutrition also impairs the development of a normal immune system during the critical periods of pregnancy, neonatal maturation, and weaning [6], [7]. Inadequate intake of dietary energy and protein leads to atrophy and alteration in the architecture of lymphoid organs, such as the thymus and spleen. Severe thymic atrophy results from massive thymocyte apoptosis (particularly affecting the immature CD4^+^CD8^+^ cell subset) and decreased cell proliferation. In the spleen, there is loss of lymphoid cells around the small blood vessels [8]–[10]. However, the influence of malnutrition on the lymph node architecture and function has not been studied.

One of the infections whose risk is increased by malnutrition is visceral leishmaniasis (VL), caused by the intracellular protozoan parasites of the *Leishmania donovani* complex (*L. infantum* (*L. chagasi*) and *L. donovani*). VL is a significant health problem in resource-poor regions of the world, particularly in India, Sudan, Bangladesh, and Brazil [11], [12]. Following inoculation of the parasite in the dermis by the bite of an infected sand fly the parasite disseminates to infect phagocytic cells of spleen, liver and bone marrow. The majority of individuals who are infected with *L. donovani* or *L. infantum* are able to control the infection and develop a sub-clinical asymptomatic infection; a minority (usually <10%) of infected individuals develops severe hepatosplenomegaly, fever, pancytopenia, and cachexia which ultimately progresses to death unless the patient is treated [13]–[16].

The factors that influence susceptibility to leishmaniasis and its progression are incompletely understood, but several lines of evidences suggest that malnutrition is a primary risk factor that contributes to the development of VL in children. Epidemiologic studies have documented an increased risk for VL in the malnourished host [2], [17]–[20] and children with moderate to severe PEM were found to have about a nine-fold increased risk of developing VL [18]. Malnutrition was identified as a risk factor for severe disease [18] and death from VL in both children (WFH<60%; OR 5.0) and adults (BMI<13; OR 11.0) [21]. Malnutrition-related VL is particularly evident in displaced and impoverished populations [22].

The mechanistic relationship between malnutrition and the course of VL at the molecular and cellular level is poorly understood. A better understanding of those mechanisms might offer new opportunities for prevention or therapeutic dietary intervention. Moreover, understanding the interplay of nutrients and immune function is of additional interest because of the malnutrition- related risk of infection with other pathogens. In previous experimental studies in a murine model of malnutrition that mimicked the growth characteristics of human weanling malnutrition [23], we observed that malnutrition caused a failure of lymph node (LN) barrier function that led to a profound increase in the early (3 days post-infection) dissemination of *L. donovani* to the visceral organs (liver and spleen). The function of the lymph node as a barrier to delay or reduce pathogen dissemination is a function of the capture of the pathogen by phagocytes (largely macrophages and dendritic cells) and restriction of its transit through the node via the size-exclusion properties of the node architecture [24], [25]. This barrier function then enables development of innate and adaptive immune responses that effect killing of the pathogen. The concept of LN barrier function has been widely discussed in the prevention of tumor metastases [26], but is less studied in the field of infectious diseases.

In the work presented here, using the murine model that we established previously [23], we further investigated the mechanisms by which polynutrient (protein, iron and zinc) deficiency (PND) impaired the capacity of the LN to act as a barrier to dissemination of *L. donovani* infection. We found that PND reduced the mass and cellularity of the LN, particularly affecting fibroblastic reticular cells and myeloid phagocytic cells, without disrupting the overall LN architecture. The function of the LN reticular conduit system was also altered and parasites were found to be associated with the conduit in the LN subcortical region and within the high endothelial venule, into which the conduits drain. The reduced number of LN phagocytes, which would affect the overall phagocytic capacity of the organ, and the altered function of the LN conduit system, therefore are likely contributors to the reduced retention and increased escape of parasites or parasitized host cells from the LN to the visceral organs.

## Materials and Methods

### Ethics statement

This study was carried out in strict accordance with the recommendations in the Guide for the Care and Use of Laboratory Animals of the National Institutes of Health. The protocol was approved by the Institutional Animal Care and Use Committee of the South Texas Veterans Health Care System where all animal experimentation was conducted.

### Experimental animals

Weanling (3-week-old) female BALB/c mice were obtained from Charles River Laboratories, Inc. (Wilmington, MA). Mice were maintained in specific pathogen-free conditions at the Veterinary Medical Unit of the Department of Veterans Affairs Medical Center, South Texas Veterans Health Care System (STVHCS), San Antonio, TX.

### Diets and feeding

Mice were initially weight-matched and housed as four mice per cage in standard polycarbonate shoebox cages with low trace element bedding (Alpha-Dri; Shepard Specialty Papers, Kalamazoo, Mich.). The mice had free access to water and were acclimatized to standard laboratory mouse chow (Teklad LM-485; Harlan Teklad, Madison, WI) for three days prior to initiation of the two experimental diets. The well-nourished (WN) control group of mice received a diet of normal mouse chow with 17% protein, 100 ppm iron, 30 ppm zinc (Teklad), which was provided ad libitum. The PND mice received a diet of mouse chow identical to the normal control diet except for low protein (3%), iron (10 ppm), and zinc (1 ppm) (Teklad), as previously described [23]. The PND mice received 90% of the weight of food consumed per day by the mice in the WN group to ensure that they did not increase their consumption in response to the nutrient deficiencies, which resulted in approximately a 10% reduction in caloric intake. Mice were fed the experimental diets until the completion of the experiment (28–31 days). The body weights of the mice were measured once per week, and food intake was recorded on a twice-weekly basis in order to calculate the amount of chow to provide to the PND group on subsequent days.

### Measurement of albumin, iron and zinc levels

Blood was collected from the mice by terminal cardiac puncture. After clotting and centrifugation the serum was collected and stored at −80°C until use. Liver tissue was collected following exsanguination of mice and stored at −80°C until use. Serum albumin levels and liver iron and zinc levels were determined by automated photometry at the Texas Veterinary Medical Diagnostic Laboratory, College Station, Texas.

### Parasites and experimental infection

*Leishmania donovani* (1S strain; MHOM/SD/00/S-2D) promastigotes were grown in complete M199 medium for 6 days and the metacyclic forms were isolated as described previously [27]. The virulence of the parasites was maintained by regular isolation from spleen tissue from infected mice or hamsters. Mice that had received the experimental diet for 28 days were inoculated with 10^6^ metacyclic promastigotes in 20 µl Dulbecco's Modified Eagle Medium (DMEM) in the skin over each hind foot-pad. In some experiments mice were infected with 2×10^6^ parasites that had been labeled with the membrane fluorescent dye PKH26 (Sigma-Aldrich, St. Louis, MO) as described previously [28].

### Quantification of parasite burdens

At 3-days post-infection, the infected mice were euthanized and the popliteal lymph nodes, spleen, and liver were harvested and weighed. Real-time quantitative PCR (qPCR) targeting kinetoplast DNA was used to quantify *L. donovani* amastigotes in the homogenized tissues as described previously [29]. Briefly, LN, spleen and liver tissues were homogenized in phosphate-buffered saline (PBS) at 1 mg/10 µl and 100 µl of the homogenate was used for DNA extraction (Qiagen DNA extraction kit). Forty ng of the extracted DNA was amplified with the Abi Prism 7900 using real-time PCR master mix kit (Applied Biosystems), 400 nM of the 13A and 13B primers [29], and 100 nM of the internal probe (5′-(6-FAM)-TTGAACGGGATTTCTGCACCCA-(TAMRA)-3′). To quantify the number of parasites, a standard curve was generated by amplification of 10-fold dilutions of *L. donovani* amastigotes isolated from the same tissue in a separate reaction. The parasite concentration was calculated per milligram of tissue, and the total organ parasite burden was calculated by multiplying this concentration by the whole-organ weight [23].

### Flow cytometry

Lymph nodes were collected in RPMI media supplemented with 2% fetal bovine serum (FBS; Gibco). Lymph node cell suspensions were prepared by cutting the tissue into small pieces and digesting it for 30 min at 37°C with collagenase D (Roche) at 2 mg/ml in buffer containing (150 mM NaCl, 5 mM KCl, 1 mM MgCl_2_, 1.8 mM CaCl_2_,10 mM Hepes pH 7.4). The tissue was further minced and strained through 100-µm cell strainers (Becton Dickinson [BD], San Jose, CA), and washed once in a solution of PBS with 2% FBS and 0.1% sodium azide. The cells were resuspended in 500 µL of RPMI with 2% FBS. The cells were counted and adjusted at a concentration from 100,000 to 500,000 cells per 50 µl, incubated for 15 min with 0.8 µg FC block at room temperature, followed by the relevant antibodies for 30 minutes at room temperature in the dark, washed again in PBS with FBS and azide and finally fixed in 250 µl of FACS lysing solution (BD, Biosciences). Cell surface analysis was performed using a combination of a panel of surface markers: FITC-conjugated rat anti-mouse Ly-6G, clone 1A8, PE-conjugated hamster anti-mouse CD11c, clone HL3, PE-conjugated rat anti-mouse Ly-6G and Ly-6C, FITC-conjugated rat anti-mouse CD11b, mouse T lymphocyte subset antibody cocktail (PE-conjugated rat anti-mouse CD4, PE-Cy7 conjugated rat anti-mouse CD3e, and FITC conjugated rat anti-mouse CD8), FITC conjugated rat anti-mouse CD45R/B220, clone RA3-6B2 (BD PharMingen, San Diego, CA), rat anti-mouse CD169, clone 3D6.112 (Abcam), FITC conjugated rat antimouse CD169, clone 3 D6.112, FITC conjugated rat anti-mouse CD31, and Alexa-Fluor 488 and 647 conjugated hamster anti-mouse CD11c, clone N418 (AbD Serotec, Raleigh, NC). For intracellular MOMA-2 analysis, cell preparations were fixed and permeabilized with fixation/permeabilization buffers (AbD Serotec) and stained with Alexa Fluor 488-conjugated rat antimouse macrophage/monocytes (AbD Serotec). For intracellular ER-TR7 analysis, cell preparations were fixed and permeabilized with a mixture of ethanol/acetone (7∶3) and stained with PE, FITC-conjugated rat antimouse ER-TR7 (Santa Cruz Biotechnology, Santa Cruz, CA), or unlabeled rat antimouse ER-TR7 (Abcam). Appropriate rat or hamster IgG isotype antibodies were used as controls. The secondary antibody APC-conjugated goat anti-rat IgG (Santa Cruz Biotechnology, inc) was used in the indirect staining. All flow cytometric analyses were performed on a FACSAria flow cytometer (Becton Dickinson, San Jose, CA, USA).

### Histopathology

The dissected popliteal lymph nodes were fixed in 10% neutral buffered formalin and processed routinely into paraffin. The fixed paraffin-embedded tissues were sectioned at 3–4 µm and stained with hematoxylin and eosin (H&E). Some paraffin embedded sections were used for reticulin stain using the method of Gomori [30].

### Electron microscopy

Popliteal lymph nodes were fixed in 4% paraformaldehyde plus 1% glutaraldehyde and processed for plastic embedment using conventional methods. Thin sections (60 to 70 nm) were stained with lead citrate and uranyl acetate. The lymph node conduit network was examined and photographed using a Jeol-JEM-1230 transmission electron microscope (Tokyo, Japan).

### Immunohistochemistry

Popliteal lymph nodes were removed from mice, immediately embedded in Tissue Tek Optimum Cutting Temperature compound (Sakura FineTek, Torrance, CA), and snap frozen and stored at −80°C until used. Sections (6 µm in thickness) were cut in a cryostat and placed on positively charged microscope slides (Superfrost/Plus, Fisher Scientific). Sections were air-dried overnight and fixed for 10 minute in ice-cold acetone. Sections were blocked with 10% serum and stained with antibodies diluted in 2% serum, which was from the same species in which the secondary antibodies were raised. Primary and secondary antibodies were applied for 60 min at room temperature in a humidified chamber. Slides were washed between and after antibody applications 5 times with PBS/0.02% BSA for 5 min each. Slides were coverslipped with Gold Prolong anti-fade mounting media (Molecular Probes, Eugene, OR). The antibodies used, and their source and specifications are summarized in Table 1. Stained lymph node sections were examined using an Olympus Provis AX 70 fluorescent microscope. The image-proplus software (Media Cybernetics, Inc., Bethesda, MD) was used to count the intensity of the fluorescence as a proportion of tissue area.

**Table 1:** Summary of antibodies used for immunohistochemistry.

Targeted mouse marker	Species	Clone	Conjugate	Vendor
CD31	Rat	GC-51	Purified	Invitrogen
ER-TR7	Rat	ER-TR7	Purified	Abcam
CD3e	Armenian hamster	145-2C11	Biotin	BD Pharmingen
MOMA-2	Rat	MOMA-2	Biotin	LifeSpan BioSciences
CD11c	Armenian hamster	N418	Biotin	Biolegend
CD11c	Armenian hamster	N418	Purified	GeneTex
CD45R/B220	Rat	RA3-6B2	FITC	BD Pharmingen
CD205	Rat	NLDC-145	Purified	Abd Serotec
CD169	Rat	3D6.112	Purified	Abcam
Collagen III	Goat	Polyclonal	Biotin	SouthernBiotech
Laminin	Rabbit	Polyclonal	Purified	Abcam
Collagen IV	Rabbit	Polyclonal	Purified	Chemicon
Actin, α-smooth muscle	Mouse	1A4	Cy3	Sigma
Collagen type I	Rabbit	Polyclonal	Purified	Chemicon
Fibronectin	Rabbit	Polyclonal	Purified	Chemicon
Heparan sulfate proteoglycan	Rat	A7L6	Purified	Abcam
Desmin	Rabbit	Polyclonal	Purified	Abcam
Rat IgG	Goat	Polyclonal	FITC	Chemicon
Rat IgG	Goat	Polyclonal	Cy3	Chemicon
Hamster IgG	Goat	Polyclonal	AMCA	Jackson ImmunoResearch
Rabbit IgG	Goat	Polyclonal	FITC	Chemicon
Rabbit IgG	Goat	Polyclonal	Cy3	Chemicon
IgG2b	Rat	Isotype Control	Biotin	Abd Serotec
IgG2a	Rat	Isotype Control	Purified	Invitrogen
IgG2a, κ	Rat	Isotype Control	FITC	BD Pharmingen
IgG	Rabbit	Polyclonal	Purified	Abcam
IgG	Armenian hamster	Isotype Control	Biotin	Biolegend
IgG	Armenian hamster	Isotype Control	Purified	Abcam
Streptavidin conjugate			Cy3	Invitrogen
Streptavidin conjugate			FITC	BD Pharmingen

### Evaluation of lymph node conduit function

For evaluation of conduit function, the skin over each footpad was injected with 20 µl low molecular weight (10 kD) or high molecular weight (500 or 2000 kD) lysine fixable Texas Red- or FITC-labeled dextran (6 mg/ml, Invitrogen, Grand Island, NY). Mice were euthanized 3 minutes after injection by CO_2_ asphyxiation and the draining popliteal lymph nodes were harvested. The lymph nodes were immediately placed in freshly prepared 4% paraformaldehyde (pH 7.4; room temperature for 1–2 h, then 4°C for 2 h), washed twice in PBS and saturated overnight at 4°C in 30% sucrose before being embedded in Tissue-Tek optimum cutting temperature compound. The sections (6 µm) were fixed in acetone for one minute and stained and visualized by fluorescence microscopy as described above.

### Statistical analysis

Data are expressed as the mean ± SEM and were analyzed using Prism software (GraphPad, La Jolla, CA). The parametric unpaired t test, or the non-parametric Mann-Whitney U test were used for the analysis depending on the normalcy of distribution of the data. Data were considered statistically significant if p≤0.05.

## Results

### Polynutrient deficient diet results in a moderate reduction in weight-for-age and decreased concentrations of biochemical nutritional parameters

**Figure 1:**
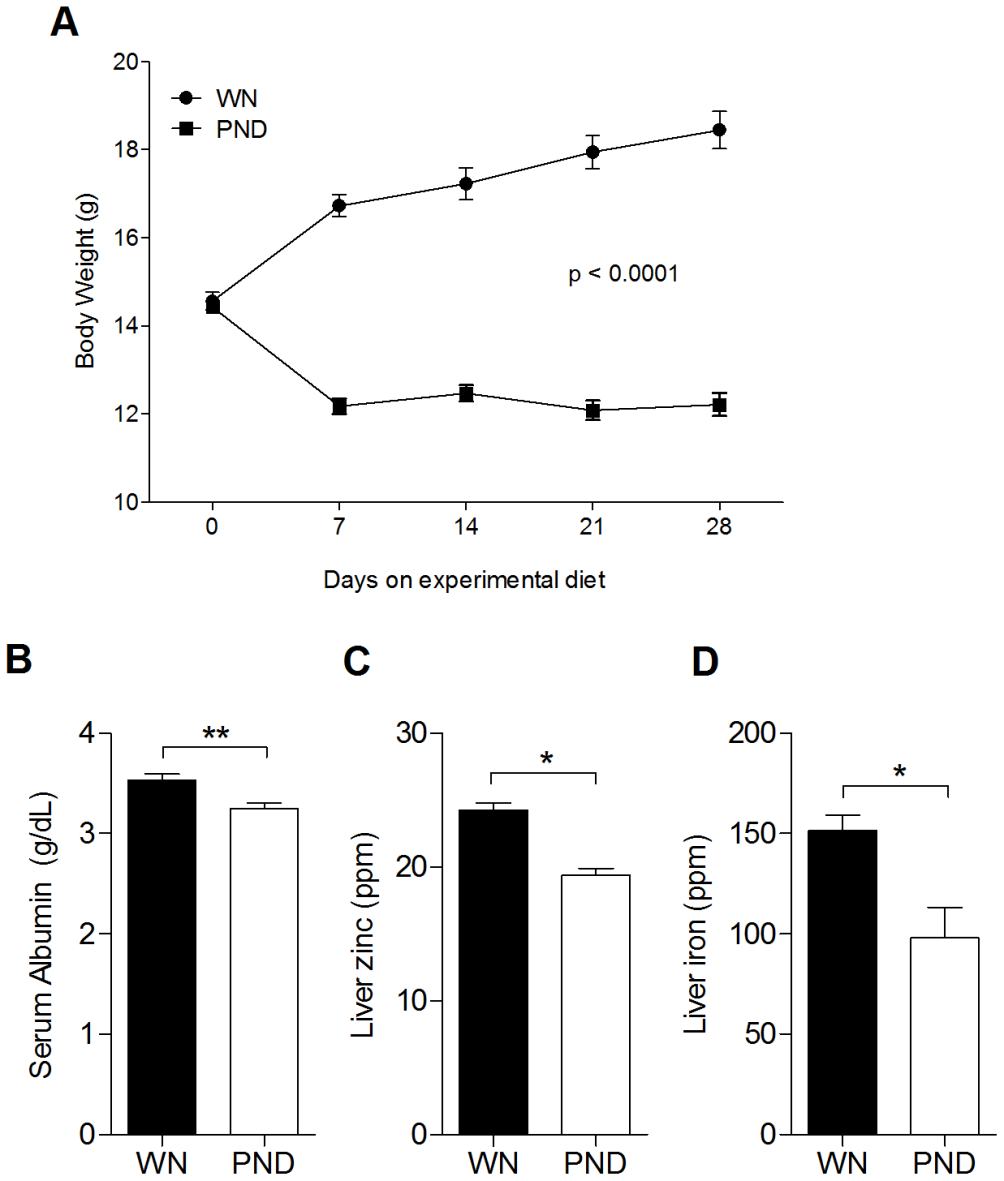
Growth curve and nutritional markers of weanling mice fed the control and the polynutrient dificient diets for 4 wks. Age-matched female weanling BALB/c mice were fed a control diet that included 17% protein, was zinc and iron sufficient, and was provided ad libitum (well-nourished; WN), or a polynutrient deficient diet (PND) that included 3% protein, was zinc and iron deficient, and had approximately a 10% caloric deficient. (A) The body weight of WN and PND mice fed with the experimental diet for four weeks. The mean and standard error of the mean (SEM; error bars) of the animal weights is shown from a single experiment (n = 8 per group) that is representative of 8 independent experiments. The significance of the difference in weights on day 28 between the WN and PND mice is shown. (B–D) Mean and SEM (error bars) of serum albumin (B), and hepatic zinc (C) and hepatic iron (D) concentrations in WN and PND mice after 28 days on the experimental diet. Data are from a single experiment (n = 6 per group for B and n = 4 per group for C and D). (*, P<0.05; **, P<0.01).

WN mice received a normal diet (17% protein, 100 ppm Fe, and 30 ppm Zn) ad libitum and consumed approximately 2.1 g of food per day. PND mice received a diet deficient in protein (3%), Fe (10 ppm), and Zn (1 ppm) and received 90% of the quantity of food consumed by the WN mice (approximately 1.9 g per day). After 4 weeks of feeding the experimental diets, the PND mice showed a slightly slumping growth curve with a 15.3% average reduction from baseline weight after 28 days (Fig. 1A), which was consistent with the previous report that showed it was comparable to moderate human weanling malnutrition [23]. To evaluate the nutritional status of the mice, the concentrations of serum albumin and hepatic zinc and iron were determined in PND mice 28 days after initiating the experimental diet. We observed a significant reduction in the serum albumin concentration (Fig. 1B; p = 0.006) and zinc and iron concentration in the liver of PND compared to WN mice (Fig. 1C, 1D; p = 0.02 and p = 0.01, respectively).

### Polynutrient deficiency leads to loss of lymph node barrier and increased early visceralization following *L.donovani* infection

**Figure 2:**
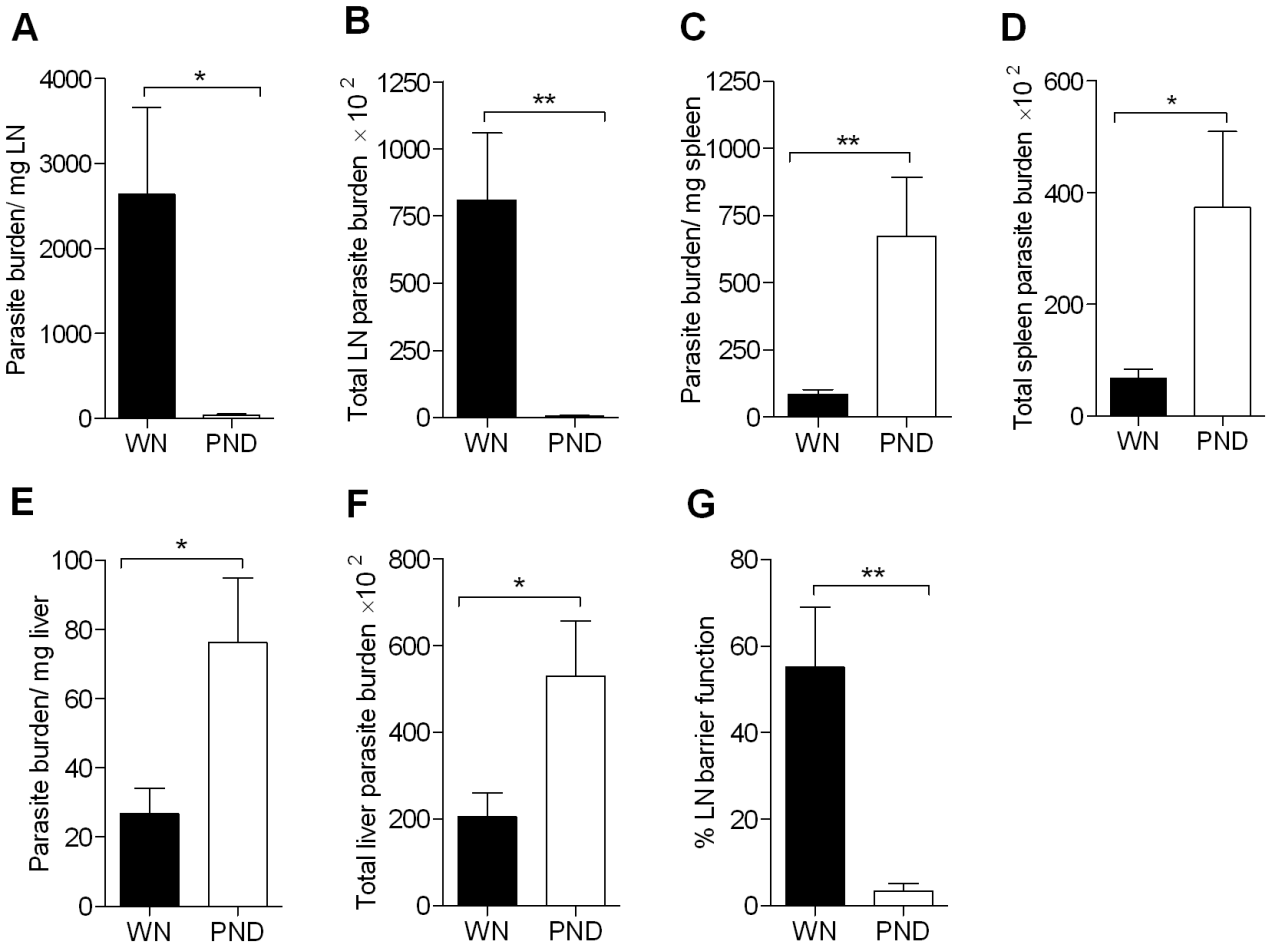
Malnutrition leads to impaired lymph node barrier function and increased early visceralization of *L.donovani*. Age-matched female weanling BALB/c mice were fed the control (well-nourished; WN) or polynutrient deficient diet (PND) for 28 days and infected with 10^6^ *L. donovani* promastigotes in the skin over each footpad. Panels A through G: At 3 days post-infection the lymph node, spleen and liver were harvested for determination of parasite burden by qPCR of parasite DNA with conversion to number of parasites by use of a standard curve. (A) Parasite burden per mg lymph node, (B) Total lymph node parasite burden, (C) Parasite burden per mg spleen, (D) Total spleen parasite burden, (E) Parasite burden per mg liver, and (F) Total liver parasite burden. (G) Calculated percent lymph node barrier function (the percentage of measured extradermal parasites retained in the lymph node) = 100−((parasite burden (PB) in liver+PB in spleen)/(PB liver+PB spleen+PB LN)) for the PND and WN mice infected with *L. donovani* for 3 days. The data shown are the mean and SEM (error bars) from a single experiment (n = 8 per group) representative of 3 independent experiments for lymph node and 6 independent experiments for liver and spleen. (*, P<0.05; **, P<0.01).

To investigate the effect of malnutrition on the early visceralization of *L. donovani*, PND and WN mice were inoculated in the skin over the hind footpad with 10^6^ *L. donovani* metacyclic promastigotes, and the parasite burdens in liver, spleen and draining (popliteal) lymph node were determined at 3 days post-infection. Consistent with our previous observations, in four different experiments using a lower parasite inoculum and shorter period of dietary deficiency than what we had described previously [23], the parasite burden in the lymph node (calculated as either the number of parasites per mg tissue or as the total organ parasite burden) was lower in the PND mice than in the WN group (Fig. 2A, 2B). Notably, the PND mice showed greater *L. donovani* dissemination to spleen (Fig. 2C, 2D) and liver (Fig. 2E, 2F) compared with the WN group. In 3 independent experiments the total measured extradermal parasite burdens (parasite burdens in LN+spleen+liver) showed no difference between the two groups of mice (for the experiment shown in Fig. 2 the total number of parasites for the WN and PND mice was 98,403±31,253 and 93,656±16,127, respectively, p = 0.9), which indicated that there was no difference in the parasite survival between the WN and the PND mice at this early stage of infection. However, the total visceral parasite burden was higher in the PND group than the WN group and the total lymph node parasite burden was higher in the WN group than the PND group, which together led to a 16-fold reduction in the calculated percent lymph node barrier function [23] in the PND infected mice (Fig. 2G). It is generally accepted that *Leishmania* traffic from the skin to the draining LN through the afferent lymphatics [31]. However, malnutrition could alter that route of transit by facilitating increased entry of the parasites directly into the bloodstream from the skin, thereby bypassing the LN. To address this possibility we quantified parasites in the skin, draining LN and visceral organs at a much earlier time point (16 hrs post-infection). We found no difference in the number of parasites in the skin or LN (Fig. S1), suggesting that malnutrition did not lead to increased visceralization by the parasite bypassing of the draining LN early in the infection process. Otherwise the parasite burden would have been reduced in both the skin and LN in the PND compared to WN mice. Collectively, these data support our previous work [23] that suggested that malnutrition produced increased visceralization after cutaneous *L. donovani* infection due to the failure of the draining lymph node to act as a barrier to dissemination.

### Polynutrient deficiency causes reduced lymph node size and cellularity

**Figure 3:**
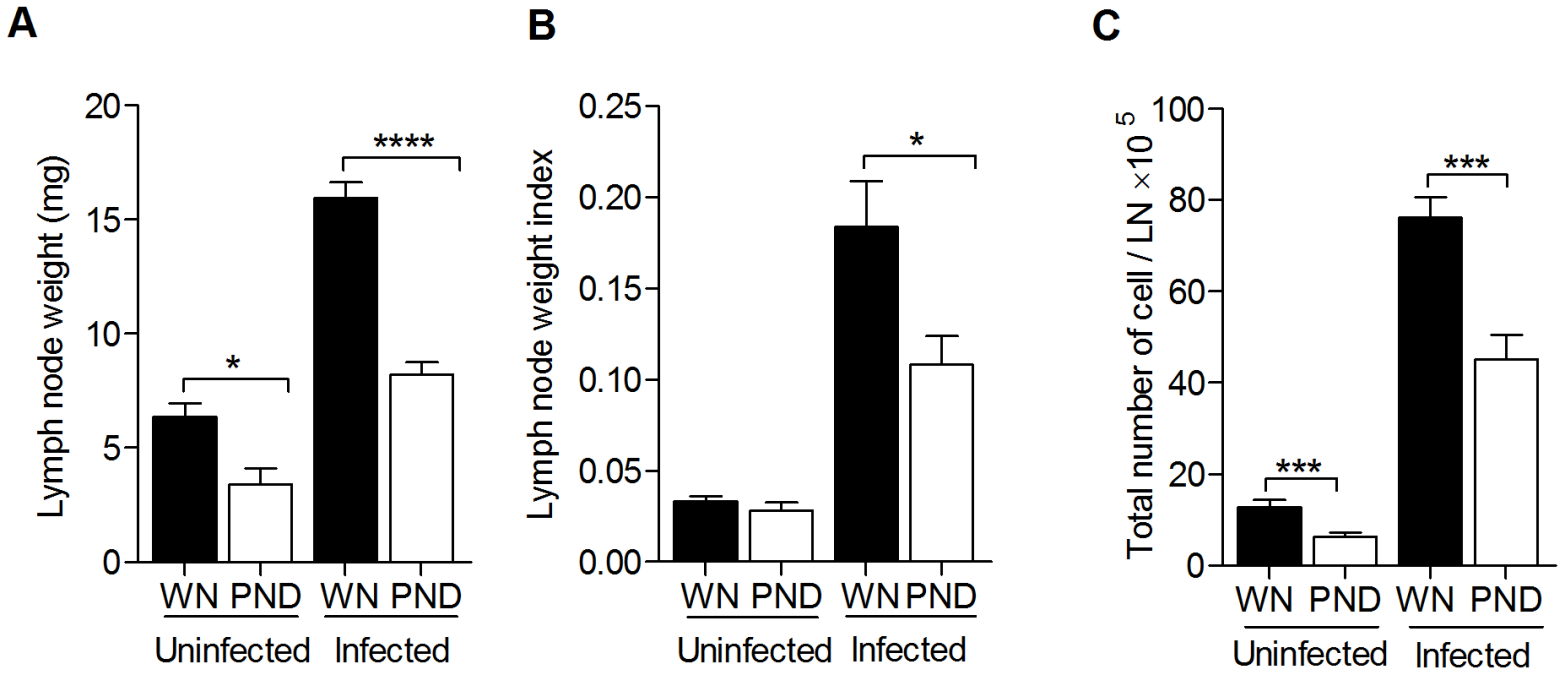
Malnutrition leads to reduced lymph node mass and cellularity. Age-matched female weanling BALB/c mice were fed the control (well-nourished; WN) or polynutrient deficient diet (PND) for 28 days and then left uninfected or infected with 10^6^ *L. donovani* promastigotes in the skin over each footpad for 3 days. The data shown are the mean and SEM (error bars) of absolute LN weight (A), LN weight index (B) and total number of LN cells (C) from a single experiment (n = 6 per group) that is representative of 3–4 independent experiments. The LN weight index was calculated as LN weight divided by bodyweight (in grams). (*, p<0.05; ***, p<0.001; ****, p<0.0001).

It was reported that lymphoid organs such as the thymus and spleen showed significant atrophy in patients with PEM or zinc deficiency [32], [33], but the effect on the LN had not been investigated. To determine the effect of polynutrient deficiency on LN mass and cellular composition, the popliteal LNs from groups of WN and PND mice were harvested before or 3 days after *L. donovani* infection. The lymph node weights were significantly less in the PND groups, whether they were infected or uninfected, compared to their WN counterparts (Fig. 3A). When corrected for body weight (LN weight index = LN weight/body weight), there was no difference in the relative weights of the LNs from the uninfected WN and PND mice, however, the LN weight index was significantly lower in the infected PND compared to infected WN mice (Fig. 3B). Consistent with the reduced LN mass we found a decrease in total LN cell number in PND compared to WN mice, regardless of whether or not they had been challenged with *L. donovani* (Fig. 3C). In both the WN and PND mice there was approximately a 6-fold increase in LN cell number at 3 days after *L. donovani* infection (Fig. 3C). Histological examination of the LN of the PND mice similarly revealed a marked decrease in the size of the LN, however, there was no obvious difference in the gross histopathology observed in hematoxylin and eosin (H&E) stained LNs of PND group compared with the WN groups (data not shown). Thus, although malnutrition caused a generalized reduction in LN mass and cellularity, it did not appear to alter the gross structure of the LN.

### Malnutrition alters lymph node cell populations in uninfected and *L. donovani* infected mice

**Figure 4:**
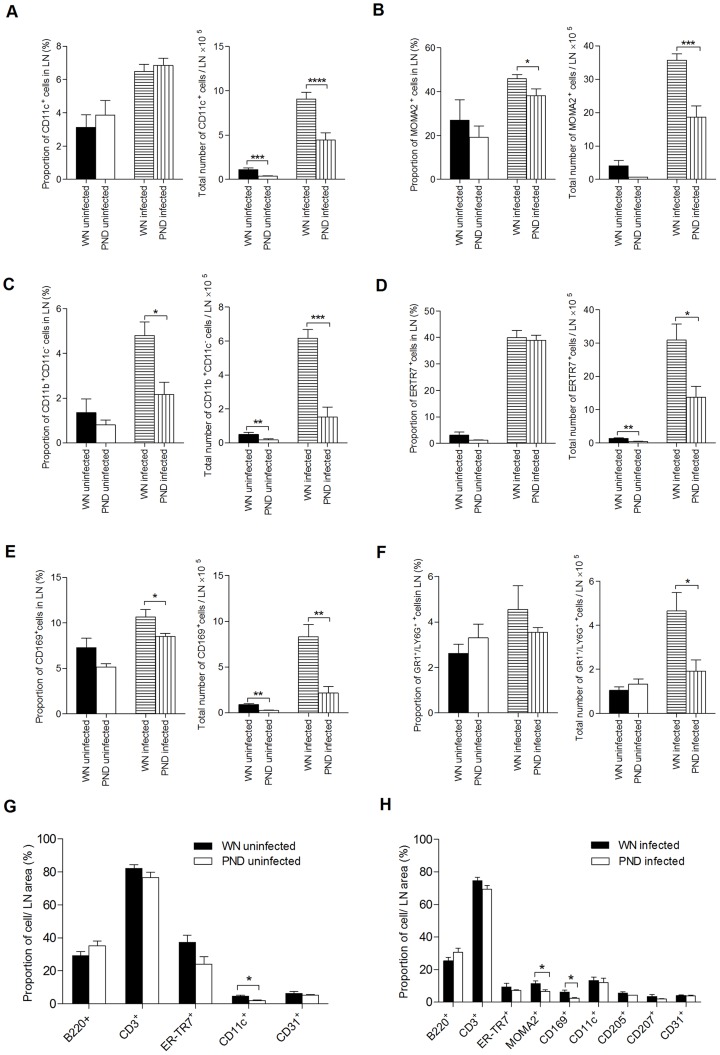
Malnutrition reduces lymph node cellularity in uninfected and *L. donovani* infected mice. Flow cytometry was used to determine the percentage (A–F, left panels) and total number (A–F, right panels) of LN cell populations in well-nourished (WN) and polynutrient deficient (PND) mice uninfected or infected with *L. donovani* for 3 days using markers for (A) DCs (CD11c), (B and C) macrophages (MOMA2 and CD11b^+^/CD11c^−^, respectively), (D) FRC (ER-TR7), (E) subcapsular sinus macrophages (CD169), and (F) neutrophils (GR1/LY6G). The different lymph node cell populations described above, as well as B cells (B220), T cells (CD3) and endothelial cells (CD31), were also quantified by immunofluorescence staining of frozen sections relative to the area of lymph node of WN and PND uninfected (G), and *L. donovani* infected mice (H). The data shown for each individual cell population in the WN and PND mice are the mean and SEM (error bars) from same experiment (n = 6 per group), which was representative of at least two independent experiments. As such, the figure includes data derived from multiple experiments since the small size of the LN in the malnourished mice precluded the quantification of multiple populations within the same LN. (*, p<0.05; **, p<0.01; ***, p<0.001; ****, p<0.0001).

To further evaluate the effect of malnutrition on the cellular composition of the lymph node, we examined cell populations in the draining lymph node of *L. donovani*-infected and uninfected WN and PND mice. We focused on the cell populations that might be involved in transporting the parasite from the site of cutaneous infection to the draining lymph node (generally ascribed to dendritic cells), as well as neutrophils, macrophages, and fibroblastic reticular cells (FRC), which may play a role in internalization of *Leishmania* in the lymph node [31], [34]. In uninfected mice, by flow cytometry we found that there was no difference in the percentage of dendritic cells (CD11c^+^) in the PND and WN groups (Fig. 4A, left panel), but the PND mice had a reduced total number of CD11c^+^ cells compared to WN controls (p = 0.0002) (Fig. 4A, right panel). This was confirmed by immunofluorescence staining of tissue sections from uninfected mice (Fig. 4G). Although we did not detect any difference in the percentage of macrophages (MOMA-2^+^ or CD11c^−^CD11b^+^), FRCs (ER-TR7^+^), subcapsular sinus (SCS) macrophages (CD169^+^ cells that line the floor of the LN subcapsular space and medulla [35]), or neutrophils (GR1^+^Ly6G^+^) between uninfected PND and WN mice (Figs. 4B–4F, left panels), the total number of CD11c^−^CD11b^+^ cells, ER-TR7^+^ cells, and CD169^+^ cells were significantly reduced in the uninfected PND compared to the uninfected WN group (Figs. 4C, 4D, 4E, right panels; p = 0.008, p = 0.008 and p = 0.004, respectively). A similar reduction in macrophages and DCs was also evident in the spleens of uninfected PND compared to WN mice (Fig. S2; p<0.001) indicating that malnutrition also had an effect on myeloid cells in organs other than the LN. No statistical difference in the total number of MOMA-2^+^ macrophages or neutrophils (Gr1^+^Ly6^+^) was found between the uninfected PND and WN groups (Figs. 4B, 4F, right panels) and immunofluorescence revealed no difference in endothelial cells (CD31^+^), B cells (B220^+^) and T cells (CD3^+^) (Fig. 4G). When draining LNs from mice challenged with *L. donovani* were examined, greater quantitative differences in the LN cell populations became evident. Independent of nutritional status, *L. donovani* infection dramatically expanded the populations of all cell types in the LN (4–47 fold-increase for WN mice and 2–75 fold-increase for PND mice; Figs. 4A–4F). In the popliteal LNs of infected PND mice compared to infected WN mice we found a reduced total number (p<0.0001) but not percentage of CD11c^+^ cells (Fig. 4A, right panel), but macrophages (MOMA-2^+^ or CD11c^−^CD11b^+^) were reduced in both percentage (p = 0.03 and p = 0.01, respectively) and total number (p = 0.0002 and p = 0.0003, respectively) in the PND infected mice (Figs. 4B and 4C). There was no difference in the percentage of FRC (ER-TR7^+^) in the infected WN and PND groups, but a significantly reduced total number of FRC was found in the PND mice (Fig. 4D; p = 0.01). CD169^+^ SCS macrophages also showed a reduced percentage and total number (Fig. 4E; p = 0.04 and p = 0.003, respectively) in the PND infected mice, and this was corroborated by immunofluorescence (Fig. 4H). The total number, but not percentage, of neutrophils (Gr1^+^Ly6^+^), was also reduced in the infected PND compared to WN mice (Fig. 4F, right panel; p = 0.02).

### Malnutrition does not alter the localization of key cell populations in the LN

**Figure 5:**
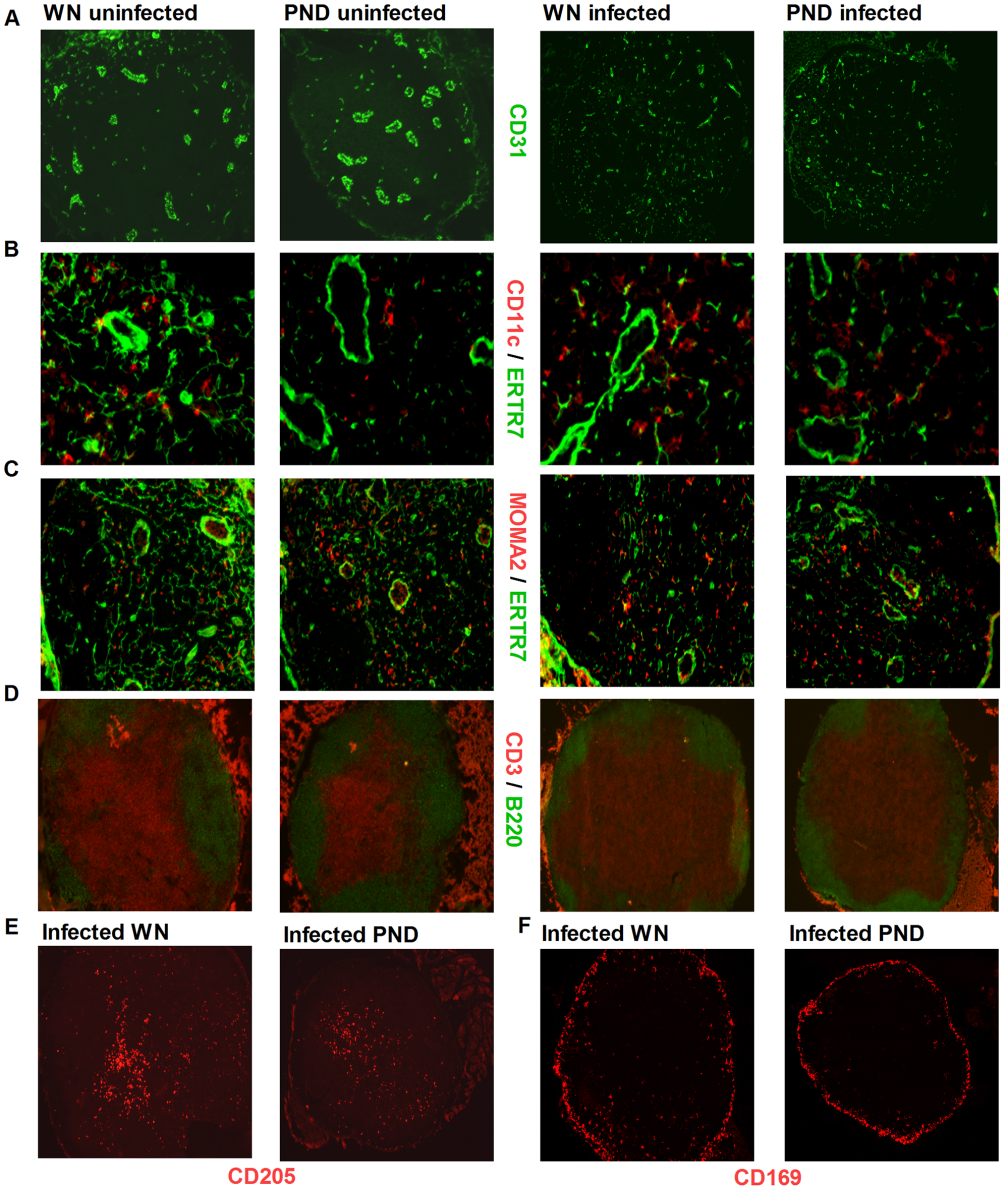
Malnutrition does not alter the cellular distribution within the lymph node. Immunofluorescence microscopy for cellular markers in popliteal lymph node cyrosections from well-nourished (WN) and polynutrient deficient (PND) mice that were uninfected or infected with *L.donovani* for 3 days using mAb against (A) endothelial cells (CD31); (B) DC (CD11c) and FRC (ER-TR7); (C) Macrophages (MOMA-2) and FRC (ER-TR7); (D) B cell (B220) and T cell (CD3); (E) Migratory Langerhans cells (CD205); and (F) Subcapsular sinus macrophage (CD169^+^). Magnification: 4× (A (infected group), D (infected group), E, F); 10× (A (uninfected group), D (uninfected group)); 20× (C); 40× (B). Images are from a single LN section from a single experiment (n = 6 per group) representative of 4 independent experiments for the infected group and one experiment for the uninfected group.

Next we investigated whether malnutrition altered the localization of different cell populations in the lymph nodes before or after *L. donovani* infection, since this would influence cell and parasite trafficking to and within the LN. Staining of serial lymph node sections for B cells, T cells, endothelial cells, macrophages/monocytes, DCs and FRC did not reveal any difference in the distribution of these cells in the LNs of PND compared to WN mice (Fig. 5A–D). In order to define the location of DCs, LN sections were co-stained for the DC marker, CD11c, and the FRC marker, ER-TR7. CD11c^+^ DCs were distributed similarly in the paracortex of all the different groups of mice. CD11c^+^ DCs associated with conduit structures were probably LN resident DC while the DCs located free in the paracortex were likely DCs that had migrated from either the dermis or conduit system (Fig. 5B). The CD11c marker does not distinguish between these cell populations. Similarly, LN sections stained for MOMA-2 and ER-TR7 showed a comparable staining pattern for macrophages/monocytes between PND and WN mice (Fig. 5C). In addition, the number of B cell follicles was comparable between the two groups (Fig. 5D) and this was consistent with the result of H&E staining (data not shown). To distinguish between LN resident DCs or dermal DCs and Langerhans cells that had migrated in response to *L. donovani* infection, we stained LN sections with antibody against CD205 [36], [37]. Consistent with the result of CD11c staining, we could not detect any difference in the distribution of CD205^+^ in the lymph nodes of WN and PND infected mice (Fig. 5E). The staining pattern of CD169^+^ cells was comparable between the PND and WN mice in the sub-capsular sinus region; however, there were more CD169^+^ cells in the cortical region of the WN infected mice (Fig. 5F). Collectively, these data indicate that while malnutrition selectively reduced the number or percentage of FRCs and several myeloid cell populations in the LN (resident and/or migratory DCs, MOMA2^+^ and CD169^+^ macrophages), it did not alter the localization of the FRCs or different phagocyte populations in the LN with the exception of reduced numbers of CD169^+^ macrophages in the sub-cortical region.

### Malnutrition alters the accumulation of small molecules within the lymph node conduit system

**Figure 6:**
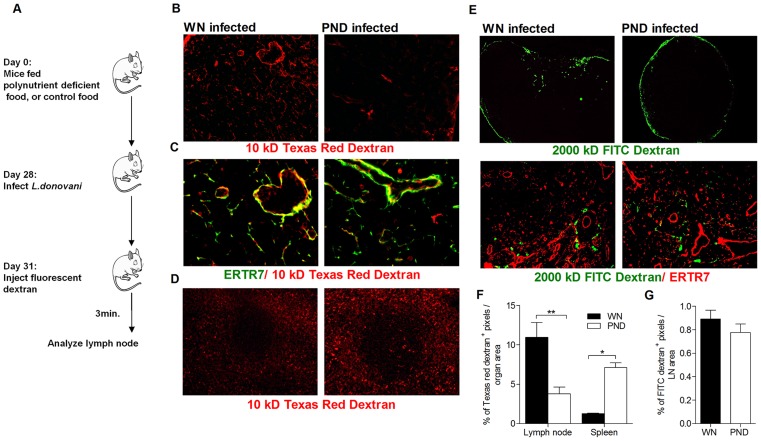
Altered LN conduit function in malnourished *L. donovani*-infected mice. (A) Schematic of approach to determine LN conduit function. Well-nourished (WN) and polynutrient deficient (PND) mice were infected with *L.donovani* for 3 days and then injected subcutaneously (both feet) with fluorescently labeled dextran. Lymph nodes were removed after 3 minutes and immediately fixed. (B) Localization of 10 kD Texas Red dextran within the lymph node. (Magnification: 20×). (C) Co-localization of 10 kD Texas Red tracer with the conduit network (FRC stained with ER-TR7). (Magnification: 40×). (D) 10 kD Texas Red dextran accumulation in the spleen. (Magnification: 20×). (E) Distribution of 2000 kD FITC dextran within the lymph node (upper panels; 4× magnification) and relative to the conduit system (lower panels; 20× magnification). (F) Quantification (mean+SEM) of 10 kD Texas Red dextran pixels as a proportion of lymph node and spleen area. (G) Quantification (mean+SEM) of 2000 kD FITC dextran pixels as a proportion of lymph node area. Data are from a single experiment with six mice per group, examining multiple sections of 2 lymph nodes per mouse, which was representative of two independent experiments. (*, p<0.05; **, p<0.01).

The stromal network has multiple functional roles in controlling the immune response in lymphoid organs by influencing cell recruitment, migration, activation, and survival. The master player in this system is the FRC, which forms a network of conduits that allows the transport of small antigens from the subcapsular sinus directly to the subcortical T cell region and the delivery of cytokines and chemokines to the port of entry of lymphocytes from the circulation, the high endothelial venule (HEV) [25], [38]. Apart from its participation in transporting the small molecules and antigens, the role of the conduit system in pathogen containment or dissemination had not been investigated. We hypothesized that malnutrition could alter the stromal architecture and conduit system to enable *Leishmania*-infected cells that have traversed the floor of the SCS to escape the LN and enter the blood stream through the HEV. To examine the influence of malnutrition on the integrity and the function of conduit system, WN and PND *L. donovani*-infected mice were injected subcutaneously with either high or low molecular weight (MW) fluorescently labeled dextran (Fig. 6A). Under normal circumstances high MW dextran is not able to traverse the floor of the SCS, whereas low MW dextran was shown to readily cross the SCS and accumulate in the LN conduit network within a few minutes after cutaneous injection [39]. In infected WN mice, Texas Red-labeled low MW dextran was clearly observed to be co-localized with the lymph node conduits 3 minutes after injection. In the PND infected mice, a reduced quantity of low MW tracer was found co-localized with the LN conduit system (Figs. 6B, 6C, 6F), but concomitantly a two-fold increase in low MW dextran accumulation was observed in the spleen (Fig. 6D, 6F), suggesting that there was impaired retention of the low MW tracer in the lymph node conduit system of the PND mice. To further assess the function and integrity of the SCS/conduit network, we injected the *L. donovani*-infected PND and WN mice with high molecular weight dextran (500 and 2000 kD). Contrary to the findings with low MW dextran, the distribution of the high molecular weight dextran was limited to the subcapsular sinus (Fig. 6E, 2000 kD shown) and medullary sinuses with little accumulation in the cortex and without co-localization with the conduit network. The distribution and quantity of the high MW dextran in the LN were comparable between the PND and WN *L. donovani*-infected mice (Fig. 6E, 6G) indicating that there was no difference in transit of the fluorescent antigen from the skin to draining LN. Collectively, these data indicate that the SCS-conduit interface was intact in PND infected mice (the high MW dextran did not gain an access to the conduit system), the integrity of the conduit was maintained without lateral leakage into the subcortical region, but the reduced retention of the small MW tracer led to greater accumulation in the spleens of PND mice.

### Malnutrition does not alter the architecture and molecular composition of lymph node conduit network

**Figure 7:**
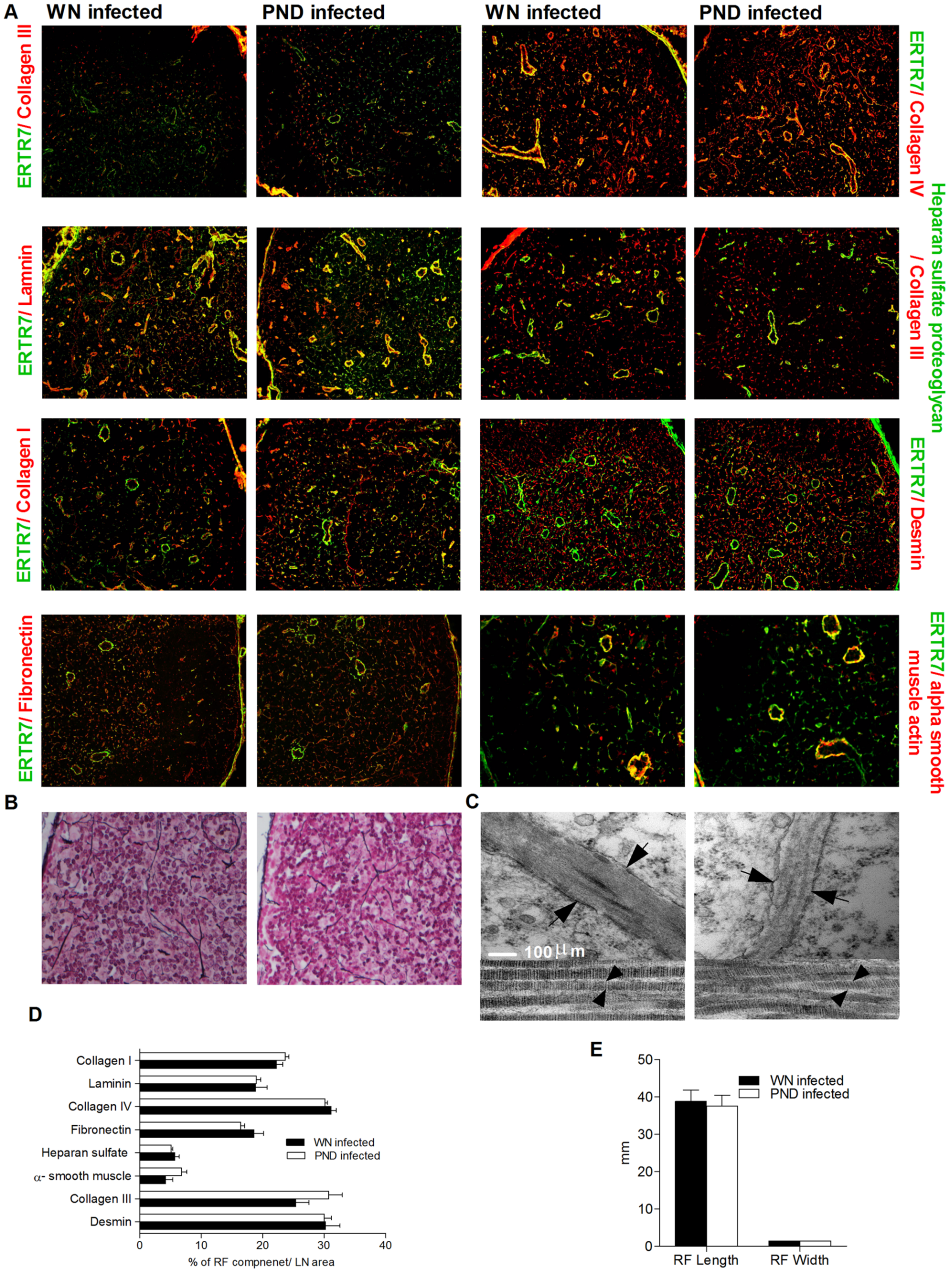
The structural framework of the conduit system is grossly intact in *L. donovani*-infected polynutrient deficient mice. Characterization of the conduit network by immunohistochemistry, reticulin stain and transmission EM in lymph nodes of well-nourished (WN) and polynutrient deficient (PND) mice 3 days after *L. donovani* infection. (A) Immunofluorescence microscopy for various components of the extracellular matrix and reticular fibers on consecutive popliteal lymph node cryosections. 20× magnification for ER-TR7 (FRC) and alpha smooth muscle and 10× magnification for all the other images. (B) Light microscopic analysis of reticular fibers visualized with the reticulin stain of paraffin embedded popliteal lymph node sections. 40× magnification. (C) Transmission electron micrographs of LN conduits in the subcortical region (upper panels; scale bar is 100 nm), arrows point to the basement membranes surrounding the conduit system, and LN conduits near the subcapsular region (lower panels; scale bar is 100 nm), arrows point to the collagen strands. (D) Quantification (mean+SEM) of pixels of each component of conduit network as a proportion of lymph node area. (E) Quantification (mean+SEM) of the length and width of the reticular fibers measured in paraffin embedded lymph node sections stained with reticulin stain. Data are from one experiment with 5 mice per group (A, D) and 4 mice per group (B, C, E). There were no statistically significant differences between WN and PND groups in panels D and E.

We investigated whether the reduced accumulation/retention of the low MW antigen in the conduit system of *L. donovani*-infected PND mice was accompanied by alteration of the architecture and the molecular components of conduit network. Staining of serial frozen sections of popliteal LNs of PND and WN infected mice with antibodies against reticular fiber components (laminin, collagen IV, heparan sulfate proteoglycan, collagen I, collagen III, fibronectin, desmin and alpha smooth muscle actin), with or without co-staining of FRC (ER-TR7 antibody), revealed no differences in the quantity or localization of any of these components in the lymph node of WN compared to PND infected mice (Fig. 7A, 7D). To further assess the structure of the LN conduit system, we measured the length and the width of the reticular fibers in LN paraffin-embedded sections following reticulin stain. Consistent with the immunohistochemistry data, we did not find any significant difference in either the length or width of the reticular fibers from the two groups of mice (Fig. 7B and 7E). Furthermore, investigation of the ultrastructure of the LN conduit system of PND and WN mice by transmission electron microscopy showed no differences in the structure of the conduit system basement membranes surrounding the collagen strands and other extracellular matrix components (Fig. 7C). Together these data suggest that the alteration of the conduit system function in PND infected mice, which allowed small molecules to escape from lymph node conduit system to the spleen, was not the result of alteration of the gross structural framework of the stroma and conduit system. By inference, and together with the finding of reduced LN phagocytic cells, the escape of the low molecular weight dextran from the conduit is likely due to the reduced phagocytic capacity (fewer DCs and macrophages) associated with the conduits.

### *L. donovani* localizes to phagocytes associated with the lymph node conduit system

**Figure 8:**
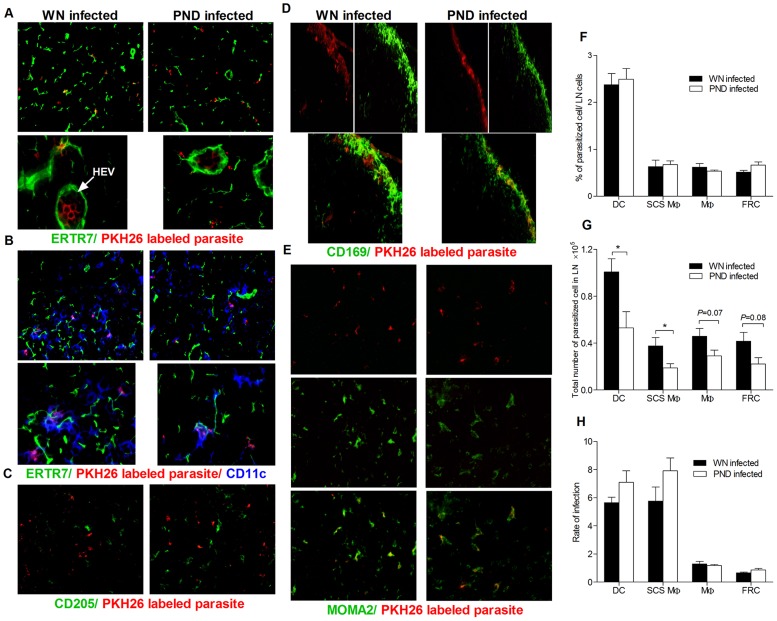
Localization of *L. donovani* in the lymph nodes of well-nourished and polynutrient deficient mice. Well-nourished (WN) and polynutrient deficient (PND) mice were infected with PKH26-labeled *L.donovani* for 3 days and the parasite localization was determined by immunofluorescence microscopy on consecutive popliteal lymph node cryosections using mAb against FRC, macrophages, and DC. (A) Parasite (red) localization with FRC labeled with ER-TR7 (green) (20× magnification). Inset shows parasites (red) inside lumen of HEV with surrounding FRC. (B) Parasite (red) co-localization with CD11c^+^ DCs (blue), with FRC labeled with ER-TR7 (green) (20× (upper panels) and 80× (lower panels) magnification). (C) Lack of parasite (red) co-localization with CD205^+^ Langerhans cells (40× magnification). (D) Parasites (red; top panel) and CD169^+^ subcapsular sinus macrophages (green; middle panel) with co-localization shown by merged images (yellow; bottom panel). (20× magnification). (E) Parasites (red; top panel) and MOMA-2^+^ macrophages (green; middle panel) with co-localization shown by merged images (yellow; bottom panel). (40× magnification). Note that these tissue sections were selected to show the localization of the parasite, and should not be used to infer relative quantity of the parasites. (F–H) Quantification of the level of cellular infection by flow cytometry. (F) Proportion of infected cell population relative to the total lymph node cells in PND and WN infected mice. (G) Total number of the infected cell population relative to the total lymph node cells in PND and WN infected mice. (H) Rate (percent) of infection of the individual cell population (proportion of the infected cell population to the total number of the same cell population). The micrographs in panels A–E are representative sections from single experiment (5 mice per group) representative of three independent experiments, and data shown in panels F–H are the mean and SEM (error bars) from a single experiment (n = 6 per group) representative of two independent experiments. (*, p<0.05).

To investigate whether there is a difference in the parasite localization within the lymph nodes of PND and WN mice, we infected mice with fluorescent-labeled *L. donovani* and examined its localization relative to FRCs (ER-TR7), macrophages (MOMA-2), DCs (CD11c), and Langerhans cells (CD205 and CD207) at 3 days post-infection and CD169^+^ cells at 2 hours post-infection. The pattern of cellular infection appeared to be similar between the PND and the WN infected mice. In both groups of mice, *L. donovani* could be observed in close association with the conduit system, but without complete co-localization with the FRCs (Fig. 8A). There was, however, a high degree of co-localization of *L. donovani* with lymph node resident DCs, and to a lesser degree with macrophages, in the vicinity of the conduit system in both PND and WN mice (Fig. 8B, E). The finding of the parasite in both groups of animals inside the high endothelial venule (HEV) (Fig. 8A) suggests that the parasite is able to transit through the conduit system, and it is likely that the reduced number of conduit-associated DCs in the PND mice enhance this transit to the systemic circulation. There was no obvious co-localization of the parasite with CD205^+^ cells (Fig. 8C) or CD207 (data not shown) Langerhans cells. There was co-localization of *L. donovani* with CD169^+^ cells in both PND and WN mice (Fig. 8D). The co-localization of the parasite with lymph node resident DC together with the parasite association with FRC indicates that the parasite might go through the conduit system despite of the size exclusion properties of the collagen III core component of the conduit network, but this did not appear to be amplified in the PND host. We used flow cytometry to further quantify the level of infection of LN cell populations and did not observe any difference in the degree of infection of macrophages or FRC between the PND and the control mice (Fig. 8H). However, there were fewer (reduced total number but not percentage) infected CD169^+^ macrophages and infected DCs in the PND mice (p = 0.04 and p = 0.02, respectively) (Fig. 8F and 8G), which probably can be attributed to the reduced total number of these cells in the PND mice (see Fig. 4D).

## Discussion

Dysfunction of the immune system is the critical link in the vicious cycle of malnutrition and infection [40], [41]. Our earlier work demonstrated that polynutrient (protein, energy, zinc and iron) deficiency led to increased dissemination of *L. donovani* from the skin to the spleen and liver, which was due to impaired barrier function and reduced parasite containment in the draining LN [23]. These studies utilized a murine model of malnutrition that mimicked the complex features of moderate childhood malnutrition found in resource-limited regions of the world, which typically involves deficiencies of protein and energy with superimposed deficits of other nutrients such as zinc [42] and iron [43]. In the work presented here, the malnutrition-related loss of LN barrier function with resulting early dissemination of the parasite was accompanied by reduced overall LN mass and cellularity, in particular reduced numbers of mononuclear phagocytes in the LN subcortical region (many of which are associated with the conduit system) and lining the floor of the subcapsular sinus. Furthermore, there were reduced numbers of FRC, which form the network of conduits that transport small molecules and antigens to the subcortical T cell regions, and there was evidence of altered conduit function. These data identify previously unrecognized effects of malnutrition on the LN and provide a foundation for understanding the early immunological events that lead to increased dissemination of *L. donovani* and perhaps other pathogens in the malnourished host.

To investigate the mechanisms of early parasite dissemination in the polynutrient deficient mice, we used intradermal inoculation of metacyclic promastigotes to mimic the natural initiation of infection by delivery of infective stage of the parasites into the skin. Distinct from our previous work, we used an earlier parasite challenge (one month after the initiation of the polynutrient deficient diet) and lower inoculum size (for more relevance to natural transmission) coupled with a more sensitive assay to quantify the parasite burden. This approach has a limitation in that the inoculum lacked the sand fly salivary components that would be included with a natural inoculation and which have been shown to promote *Leishmania* infection [44] and could enhance the dissemination of parasites from skin to the viscera. Nevertheless, we found about a 16-fold reduction in the percent of lymph node barrier function, and conclude that the early parasite dissemination is the result of the impaired capacity of the lymph node to contain the parasite locally. Since the total extradermal parasite burdens (local draining lymph nodes, spleen, and liver) were comparable between the two groups, it appears unlikely that early increase in the parasite visceralization was due to defective local parasite killing in the lymph node (although this is likely to be an issue later in the course of infection in the malnourished host [45]) or a difference in the rate of the parasite multiplication. Furthermore, hematogenous dissemination of *L. donovani* from the site of skin infection is not likely to contribute significantly to the malnutrition-related parasite visceralization because the parasite burdens in the skin and draining LN were no different in the WN and PND mice at an early time point.

While the effect of malnutrition on the LN has not been described previously, a number of malnutrition-related changes in the composition and structure of other lymphoid tissues have been reported, including (1) atrophy of thymus and spleen [8], [32], [46], (2) reduced thymic cellularity attributed to enhanced thymocyte apoptosis and decreased intrathymic cell proliferation [47], [48], (3) alteration in the thymic microenvironment [49], [50], (4) reduced in vivo and in vitro bone marrow cell proliferation [51], [52], (5) loss of splenic lymphoid cells around the small blood vessels [8], and (6) reduced number of splenic T lymphocyte subsets [53]. We did not find any remarkable difference in the gross structure or cellular distribution within the lymph nodes, however, consistent with the previous observations in the thymus and spleen we did observe a significant reduction in the weight and cellularity of the LN of the PND mice, whether they were uninfected or infected with *L. donovani*, when compared with their WN controls. Myeloid populations within the lymph node were most significantly affected by PND. Uninfected PND mice had fewer LN dendritic cells compared with the WN controls, but following infectious challenge reduction in LN dendritic cells, macrophages and neutrophils was evident. These findings, along with the 2-fold reduction in the number of the parasitized LN DCs, in the infected PND mice suggest that malnutrition contributes to parasite dissemination through several possible mechanisms. First, the reduced numbers of resident DCs and macrophages in the LN may lead to overwhelming of the phagocytic capacity of the organ with escape of parasites to the systemic circulation and visceral organs. The lower retention of the low molecular weight tracer within the lymph node conduit system and increased trafficking to the spleen in the PND infected, probably the result of reduced phagocytic capture, mice supports this possibility of increased dissemination of the parasite through the conduit system. Second, malnutrition may lead to altered migration and/or LN retention of parasitized DCs leading to increased parasite dissemination. In support of the later, it is commonly held that dendritic cells are the primary means by which *Leishmania* is transported from the site of skin infection to the lymph node [54], and some studies have also implicated macrophage in this process [31]. We could not detect any co-localization of the parasite with CD205^+^ or CD207^+^ cells, which indicates that Langerhans cells do not play a role in moving the parasite to the draining lymph node. This is consistent with recent observations in another *Leishmania* infection model [54]. Altered DC migration and maturation, cytokine production, and adhesion molecule expression was demonstrated previously in human malnutrition [55]. This impaired DC function may be related to reduced leptin levels [56], [57], and/or increased levels of prostaglandin E_2_ [58], both of which were found in earlier work to be abnormal in our model ([23], [59], and GM Anstead, unpublished data), and therefore could play a role in altered migration of *Leishmania*-infected DCs in the PND host. The route through which infected DCs might disseminate is currently under investigation.

Subcapsular sinus (CD169^+^) macrophages, which line the floor of the subcapsular sinus and medulla of the lymph node and play a key role in the lymph filtration and the translocation of the large or particulate antigens across the sub capsular sinus lining to the cortex [60], were reduced in the infected PND mice. A recent study showed that depletion of CD169^+^ cells led to dissemination of vesicular stomatitis virus through the lymphatics after subcutaneous inoculation of mice with the virus [35]. The reduced numbers of CD169^+^ macrophages in the infected PND mice may lead to impaired transmigration of the parasite to the LN cortical region and thus favor transit of parasites from the subcapsular sinus directly to the efferent lymph and dissemination to the bloodstream.

The LN reticular network plays crucial functional and structural roles in the defense against pathogens by promoting interaction between T cells and antigen-presenting cells, enabling rapid transport of free antigens through the conduit system for uptake and presentation by resident DCs to T cells [25], [61], and helping in the recruitment, retention and proper localization of immune cells. FRCs establish the reticular network by secreting extracellular components to produce reticular fibers which are interweaved to form the conduit system [62], [63]. It was reported that the FRC is a target cell during infection by multiple pathogens, particularly those that persist chronically, including *L. major* infection [34]. Our data showed that *L. donovani* was associated with the conduit, and was found co-localized with the resident DCs surrounding it, in both WN and PND mice. We did not identify infected FRC but found the numbers of FRCs were decreased significantly in the *L. donovani* infected PND mice. Since FRCs produce DC chemoattractants such as CCL19 and CCL21 [64], the decreased number of FRCs may significantly alter chemoattraction and retention signals, possibly resulting in increased escape of parasite-loaded phagocytes from the lymph node to the visceral organs.

Lymph flows from the afferent lymphatics into the lymph node subcapsular sinus, then to the conduit system and out through HEV into the bloodstream [39]. The presence of the parasite in association with the conduit system and in the HEV suggests that they may traverse the conduit system into the HEV to disseminate through the blood stream. Furthermore, the presence of the parasite in the lymph node very early in the infection suggests that the parasite may be carried through the lymph and enter subcortical region via the conduit network independent of migratory DCs. Evidence supporting this idea comes from the previous work that demonstrated activation of lymph node resident DCs surrounding the conduit system within a few hours of *L. major* inoculation, while migration of skin derived DCs to the LN was not evident until approximately 14 hours after infection [37], [61]. Additionally, *L. chagasi* was found in the draining lymph node of infected hamster two hours after infection [65]. Since the LN conduit network allows only small molecules (<70 kD) to pass along the reticular fibers [25], [39], [66], [67], we suspected that there might be a breach in the integrity of the floor of the SCS allowing entry of the much larger parasites into the conduit system. However, we found the high molecular weight dextran was retained in the subcapsular sinus without association with the FRC network indicating that a functional barrier was intact. This suggests that parasite trafficking through the LN is an active process, perhaps mediated by transmigrating subcapsular sinus macrophages or migratory DCs, but it remains to be determined how the parasite escapes the size exclusion property of the reticular fiber to gain an access to the conduit system. The presence of comparable quantities of the high molecular weight dextran in the LNs of PND and WN mice indicates that the influx of the tracer from the skin to the LN was not altered in the PND mice and that the reduced amount of the low molecular weight dextran in the conduit system of PND mice was likely to be due altered transmigration and/or retention.

In summary, to our knowledge this study is the first to describe the architecture and cellular composition of the lymph node in the malnourished host. Based on our findings, four possible scenarios could explain how malnutrition leads to the loss of lymph node barrier function and early dissemination of *L. donovani*. First, the reduced total number of DCs and macrophages (in both the subcapsular sinus and subcortical regions), with the resulting decrease in numbers of parasitized cells in the lymph node of the PND mice, would translate to a reduction in overall phagocytic capacity of the lymph node as an organ and allow the escape of parasites. Second, the reduced number of CD169^+^ macrophages may lead to impaired parasite capture and transmigration of the infected phagocyte into the lymph node cortical region allowing the parasite to escape the lymph node through the efferent lymphatic to the bloodstream and the visceral organs. Third, the reduced number of LN DCs may also alter trafficking and/or reduced retention of parasitized DCs in the LN. Lastly, the altered function of the LN conduit system, which may be related to a deficiency in resident macrophages and DCs along the conduit system resulting in reduced capture of parasites as they transit through the conduit, could lead to increased dissemination through the HEV to the systemic circulation. While there is support for each of these scenarios from the data presented here, and they are not mutually exclusive, further work is warranted to clearly define the route and mechanisms of visceralization in the malnourished host.

## Supporting Information

Figure S1 **Trafficking of** ***L. donovani*** **from the skin to the lymph node early after infection.** Age-matched female weanling BALB/c mice were fed the control (well-nourished; WN) or polynutrient deficient diet (PND) for 28 days and infected with 10^6^ *L. donovani* promastigotes in the skin over each footpad. At 16 hours post-infection the lymph node, spleen, liver and footpad tissue were harvested for determination of parasite burden by qPCR of parasite DNA with conversion to number of parasites by use of a standard curve. (A) Total parasite burdens in lymph node, spleen, and liver. (B) Parasite burden per mg lymph node, spleen, and liver. (C) Parasite burdens calculated per mg footpad tissue and total footpad parasite burden. The data shown are the mean and SEM (error bars) from a single experiment (n = 8 per group). (** p<0.01).
(TIFF)

Figure S2 **Malnutrition reduces the numbers of macrophages and dendritic cells in the uninfected spleen.** Flow cytometry was used to determine the percentage (A) and total number (B) of macrophages (CD11b^+^/CD11c^−^) and DCs (CD11c^+^), in well-nourished (WN) and polynutrient deficient (PND) uninfected mice. The data shown are the mean and SEM (error bars) from a single experiment (n = 7 per group). (**, p<0.01; ***, p<0.001; ****, p<0.0001).
(TIF)
