# Supplementary figures and images for: The Malnutrition-Related Increase in Early Visceralization of Leishmania donovani Is Associated with a Reduced Number of Lymph Node Phagocytes and Altered Conduit System Flow
Source: PLoS Negl Trop Dis. 2013 Aug 15;7(8):e2329. doi: 10.1371/journal.pntd.0002329 (PMC3744437; doi:10.1371/journal.pntd.0002329)

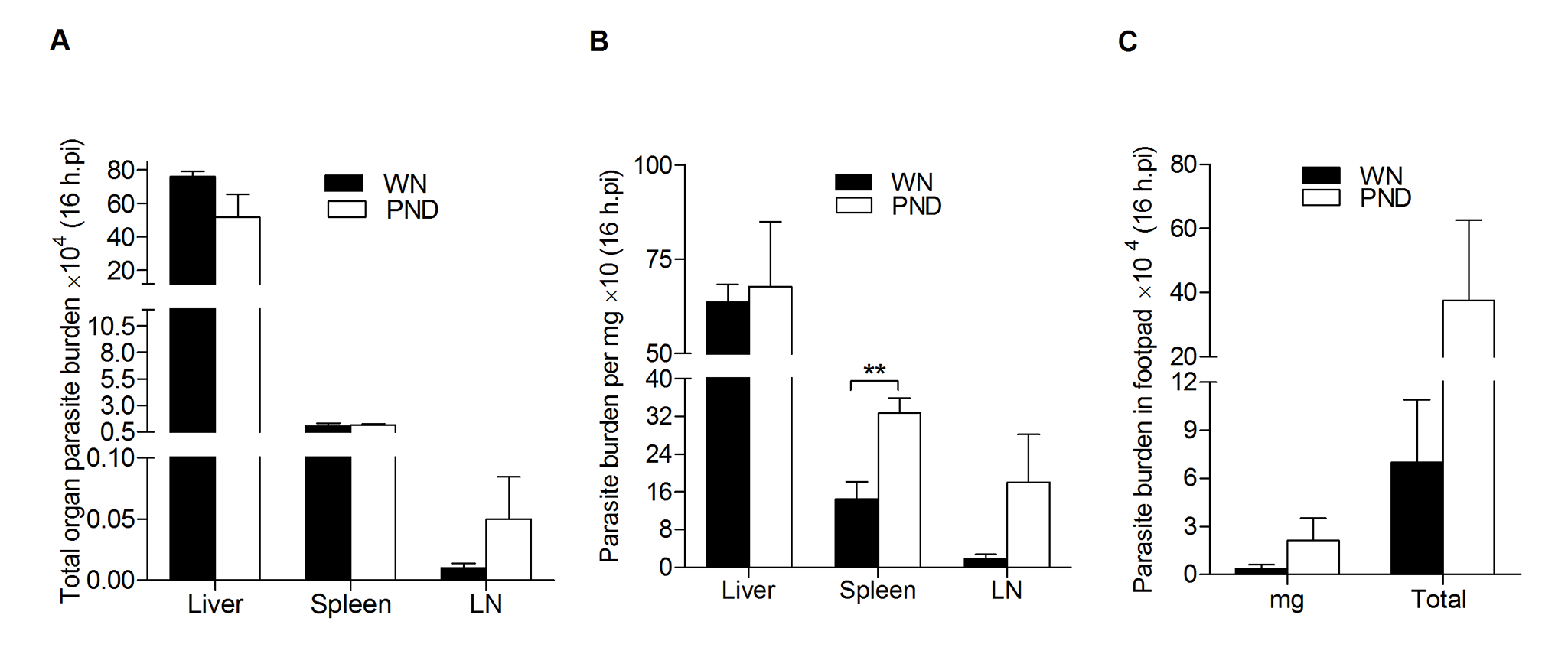

Supplement: Figure S1 — Trafficking of L. donovani from the skin to the lymph node early after infection. Age-matched female weanling BALB/c mice were fed the control (well-nourished; WN) or polynutrient deficient diet (PND) for 28 days and infected with 106 L. donovani promastigotes in the skin over each footpad. At 16 hours post-infection the lymph node, spleen, liver and footpad tissue were harvested for determination of parasite burden by qPCR of parasite DNA with conversion to number of parasites by use of a standard curve. (A) Total parasite burdens in lymph node, spleen, and liver. (B) Parasite burden per mg lymph node, spleen, and liver. (C) Parasite burdens calculated per mg footpad tissue and total footpad parasite burden. The data shown are the mean and SEM (error bars) from a single experiment (n = 8 per group). (** p<0.01). (TIFF) [file pntd.0002329.s001.tiff]

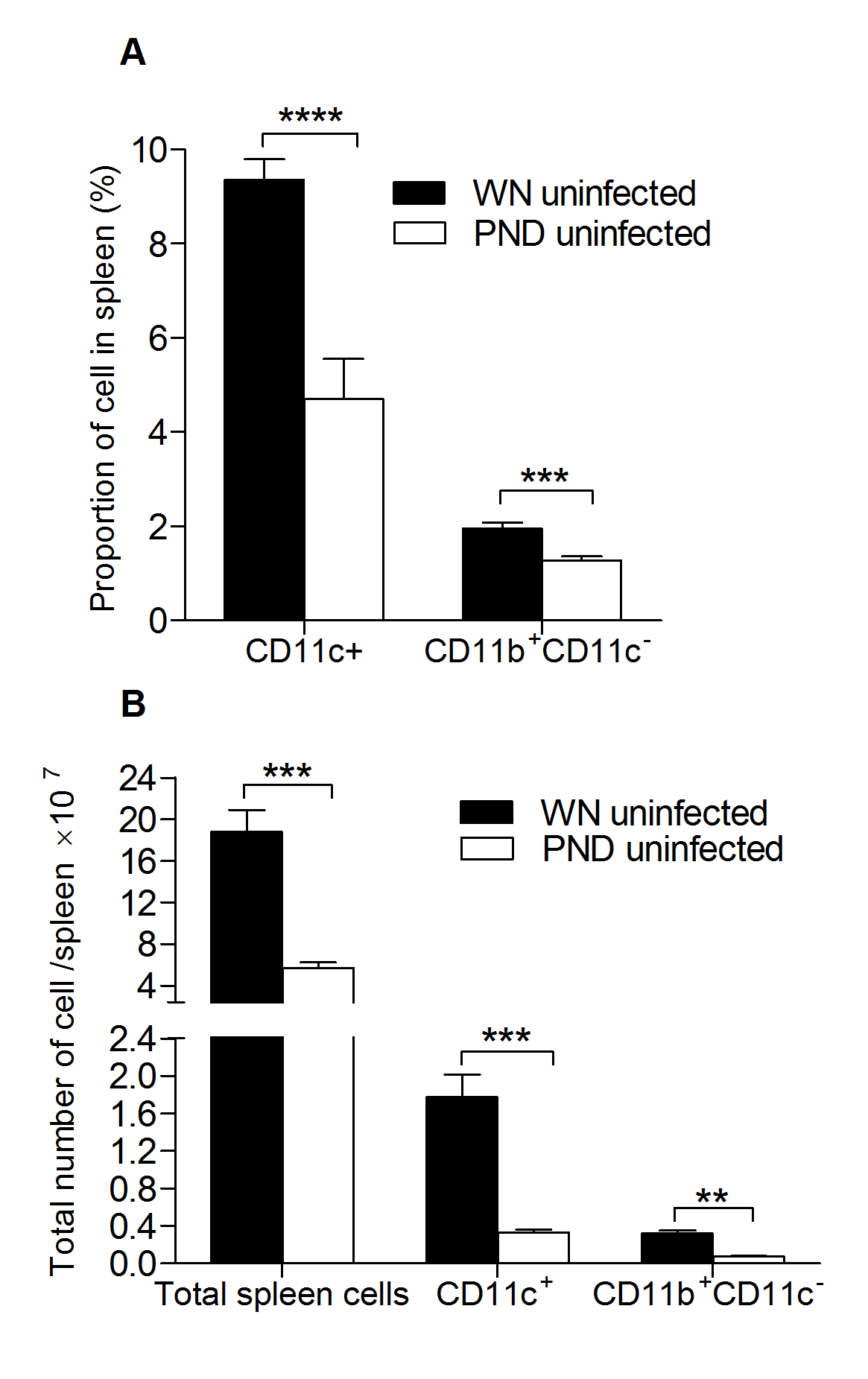

Supplement: Figure S2 — Malnutrition reduces the numbers of macrophages and dendritic cells in the uninfected spleen. Flow cytometry was used to determine the percentage (A) and total number (B) of macrophages (CD11b+/CD11c−) and DCs (CD11c+), in well-nourished (WN) and polynutrient deficient (PND) uninfected mice. The data shown are the mean and SEM (error bars) from a single experiment (n = 7 per group). (**, p<0.01; ***, p<0.001; ****, p<0.0001). (TIF) [file pntd.0002329.s002.tif]
